# CXCR2 antagonist for patients with chronic obstructive pulmonary disease with chronic mucus hypersecretion: a phase 2b trial

**DOI:** 10.1186/s12931-020-01401-4

**Published:** 2020-06-12

**Authors:** Aili L. Lazaar, Bruce E. Miller, Alison C. Donald, Thomas Keeley, Claire Ambery, John Russell, Henrik Watz, Ruth Tal-Singer, for 205724 Investigators, Philip Bardin, Peter Bremner, David Langton, Anne-Marie Southcott, Paul S. Thomas, John Wheatley, Kenneth R. Chapman, Murdo Ferguson, Lawrence A. Homik, Francois Maltais, Bonavuth Pek, Eric St-Amour, Tamara Eckermann, Andreas Eich, Guido Ern, Karin Foerster, Andreas Forster, Martin Hoffmann, Claus Keller, Anneliese Linnhoff, Ruth Nischik, Isabelle Schenkenberger, Olaf Schmidt, Joong Hyun Ahn, Hee Soon Chung, Do-Jin Kim, Jae Yeol Kim, Sang Haak Lee, Yeon-Mok Oh, Myung Jae Park, Suk Joong Yong, Simone Van der Sar, Pascal L. M. L. Wielders, Anna Olech-Cudzik, Krzysztof Wytrychowski, Ghiulten Apti, Andreia Madalina Balta, Doru Didita, Livia Filip, Bogdan Mihai Mincu, Viorica Mincu, Roxana Maria Nemes, Maria Elena Scridon, Antigona Carmen Trofor, Dragos G. Ungurean, Ramon Agüero Balbín, Miguel Barrueco Ferrero, José Maria Echave-Sustaeta, José María Marín Trigo, Eduardo Monso Mola, Sergi Pascual Guardia, Germán Peces-Barba Romero, Roger A. Abrahams, Thomas M. Hyers, Edward M. Kerwin, Shawn M. Magee, Murali Ramaswamy, James Michael Wells

**Affiliations:** 1 GSK, Collegeville, PA USA; 2 GSK - Stockley Park, Uxbridge, UK; 3 Pulmonary Research Institute Lungen Clinic Grosshansdorf, Airway Research Center North (ARCN), German Center for Lung Research (DZL) –, Grosshansdorf, Germany

**Keywords:** COPD, Clinical trial, Danirixin, CXCR2

## Abstract

**Background:**

Oral CXC chemokine receptor 2 (CXCR2) antagonists have been shown to inhibit neutrophil migration and activation in the lung in preclinical and human models of neutrophilic airway inflammation. A previous study with danirixin, a reversible CXCR2 antagonist, demonstrated a trend for improved respiratory symptoms and health status in patients with COPD.

**Methods:**

This 26-week, randomised, double-blind, placebo-controlled phase IIb study enrolled symptomatic patients with mild-to-moderate COPD at risk for exacerbations. Patients received danirixin 5, 10, 25, 35 or 50 mg twice daily or placebo in addition to standard of care. Primary end-points were the dose response of danirixin compared with placebo on the incidence and severity of respiratory symptoms (Evaluating Respiratory Symptoms in COPD [E-RS:COPD] scores) and safety. Secondary end-points included the incidence of moderate-severe exacerbations, health status (COPD Assessment test, CAT) and health-related quality of life HRQoL (St. George Respiratory Questionnaire-COPD, SGRQ-C).

**Results:**

A total of 614 participants were randomized to treatment. There were no improvements in E-RS:COPD, CAT or SGRQ-C scores in participants treated with any dose of danirixin compared to placebo; a larger than expected placebo effect was observed. There was an increased incidence of exacerbation in the danirixin-treated groups and an increased number of pneumonias in participants treated with danirixin 50 mg.

**Conclusions:**

The robust placebo and study effects prohibited any conclusions on the efficacy of danirixin. However, the absence of a clear efficacy benefit and the observed increase in exacerbations in danirixin-treated groups suggests an unfavorable benefit-risk profile in patients with COPD.

**Trial registration:**

This study was registered with clinicaltrials.gov, NCT03034967.

## Background

The inflammation associated with Chronic Obstructive Pulmonary Disease (COPD) is characterized by a prominent infiltration of neutrophils in lung tissue and the airways [1]. There is a large body of evidence that the CXC chemokine receptor 2 (CXCR2) on the neutrophil plays a pivotal role in neutrophil recruitment to the lung. Oral CXCR2 antagonists have been shown to inhibit neutrophil migration and activation in the lung in preclinical and human models of neutrophilic airway inflammation, as well as in established neutrophilic airway diseases [2–6].

The selective CXCR2 antagonist GSK1325756/danirixin (DNX) has demonstrated potent antagonism of CXCR2 activity both in vitro and in vivo in preclinical studies [6]. In a recent Phase II study, danirixin reduced respiratory symptoms, as measured by the Evaluating Respiratory Symptoms in COPD (E-RS: COPD) patient reported outcome (PRO) tool, in symptomatic COPD patients who were at risk of exacerbation [7]. The primary objectives of the current study were to evaluate the dose response of DNX compared with placebo on respiratory symptoms assessed by E-RS: COPD and assess the safety of DNX compared with placebo. Key secondary objectives included an evaluation of DNX compared with placebo on healthcare resource utilization (HCRU) defined COPD exacerbations, health status and rescue medication use.

## Methods

### Study design and objectives

Between April 2017 and October 2018, we conducted a phase II study in 64 centers in 9 countries (GSK protocol 205,724; ClinicalTrials.gov identifier NCT03034967). The study protocol, any amendments, the informed consent, and other information that required pre-approval were reviewed and approved by a national, regional, or investigational center ethics committee or institutional review board. The study was conducted in accordance with the revised Declaration of Helsinki (2008), International Council for Harmonisation guidelines for Good Clinical Practice, and all applicable regulatory requirements. Full written informed consent was obtained from all participants before the performance of any study-specific procedures.

Consenting males or females, aged 40–80 years of age with a current diagnosis of COPD based on American Thoracic Society/European Respiratory Society guidelines [8] (postbronchodilator forced expiratory volume in 1 second [FEV1]/forced vital capacity [FVC] ratio < 0.7 and FEV1% predicted ≥40% [9]) and with a smoking history of ≥10 pack years were eligible to participate in the study. Eligible participants were required to have a history of respiratory symptoms including chronic cough, mucus hypersecretion, and dyspnea on most days for at least the previous 3 months prior to screening, and a documented history of COPD exacerbations in the 12 months prior to study participation (≥2 moderate-severe exacerbations or 1 moderate-severe exacerbation plus a screening plasma fibrinogen concentration of ≥3 g/L [10–12]). Full inclusion and exclusion criteria are described in the online supplement.

The study was a randomised, double-blind, placebo-controlled, parallel-group study. Following a screening visit and completion of the run-in period assessments, participants were randomised (1:1:1:1:1:1) using an interactive voice response system to receive oral danirixin hydrobromide salt tablets (5, 10, 25, 35 or 50 mg) or placebo tablets for 24 weeks (in addition to COPD standard-of-care). The dose range used was based on a relative bioavailability study comparing two formulations of danirixin, which demonstrated that the danirixin hydrobromide salt has approximately twice the bioavailability of danirixin free base (used in the previous Phase 2a study) [13]. The dose range tested would allow estimation of the danirixin dose-response curve; the top dose of 50 mg was chosen to avoid exposures that could exceed the safety margin required based on non-clinical safety assessment studies.

The primary endpoints were safety (adverse events (AEs), vital signs, 12-lead electrocardiogram, clinical laboratory and hematological evaluations) and the change from baseline in respiratory symptoms measured by the E-RS: COPD daily diary at month 6, both total score and subscales (i.e., breathlessness, cough and sputum, and chest symptoms). Secondary endpoints included HCRU-defined COPD exacerbations, time to first HCRU-defined COPD exacerbation, number of EXAcerbations of Chronic Pulmonary Disease Tool (EXACT) tool defined events, change from baseline in the St. George’s Respiratory Questionnaire (SGRQ) total score (derived from St. George’s Respiratory Questionnaire – COPD specific [SGRQ-C]), change from baseline of the COPD Assessment Tool (CAT) total score, lung function (FEV1, FEV1% predicted, FVC, FEV1/FVC ratio), rescue medication use, participant experience of physical activity (subset of participants) measured using Clinic Visit PROactive Physical Activity in COPD (CPPAC), and pharmacokinetics. Biomarker assessments included measurements of systemic inflammation (i.e., C-reactive protein [CRP] and fibrinogen), and markers of extracellular matrix turnover.

### Assessment methodology

Respiratory symptoms were evaluated using the E-RS: COPD, an 11-item, patient-reported outcome instrument completed each evening using an electronic diary (eResearch Technology, Inc. [Philadelphia, PA, USA]) as part of the 14-item EXACT [14, 15], which was also measured as part of the study. The E-RS: COPD yields a total score, quantifying respiratory symptom severity overall, with a score range of 0–40 and higher scores indicating more severe symptoms. It has been suggested that score changes ≥2 are clinically meaningful [14, 15]. Three domain or subscale scores assess breathlessness (scores 0–17, meaningful change 1.0 point), cough and sputum (0–11; meaningful change 0.7) and chest symptoms (0–12; meaningful change 0.7). Monthly weighted mean scores for E-RS: COPD total and domain scores were calculated. Baseline scores were defined as the average score over days 1–7 of the run-in period. Responders were defined as those with a change from baseline equal to or greater than the minimal clinically important difference (MCID).

Symptom-defined exacerbations were identified using the EXACT [16]. This instrument assesses the severity of respiratory and systemic manifestations of a COPD exacerbation as reported by the patient to capture the occurrence, frequency, severity, and duration of symptom-defined events. The EXACT total score ranges from 0 to 100; higher scores indicate more severe symptoms, with sustained worsening > 9 points for 3 days or 12 or more points for 2 days constituting the onset of a symptom-defined event.

COPD-related health status was assessed during clinic visits at days 1, 84 and 168 and follow-up using the 8-item CAT questionnaire [17]. Patients rated their experience on a 6-point scale, where 0 = no impairment and 5 = maximal impairment, summed to yield a total score range of 0–40. Higher scores indicate greater disease impact. Responders were defined as patients with health status improvement indicated by a decrease from baseline in CAT score of ≥2 [18].

The SGRQ-C is an FDA-qualified, COPD disease-specific questionnaire derived from the SGRQ, designed to measure the impact of respiratory disease and its treatment on a COPD patient’s HRQoL [19]. The SGRQ-C comprises of 40 questions, and total score and MCID are equivalent to the SGRQ instrument [20]. Responders were defined as those with a decrease from baseline equal to or greater than the MCID, defined as a 4-point improvement (decrease) [21].

Actigraph GT9X activity monitors were provided by Actigraph (Pensacola, FL, US), and issued to a subset of consenting participants for physical activity monitoring. Methods for assessment of PK concentrations and biomarkers were as previously described [22–24].

### Statistical analyses

The sample size estimations were based on the primary efficacy endpoint of change from baseline in respiratory symptoms measured by E-RS: COPD daily diary at month 6. Based on simulations, a sample size of at least 600 participants (100 in each of the 6 study treatment groups) was used to allow for adequate precision in estimation of the 35 mg DNX dose as well as a sufficient proportion with 90% confidence interval (CI) difference from placebo excluding 0, and a sufficient proportion with 90% CI of dose estimate excluding 0.

Three interim analyses were planned and performed for this study: 1) The first interim analysis was an evaluation of DNX PK conducted after 10 participants in each treatment group had participated in the PK sub-study; 2) an interim analysis for futility based on the E-RS: COPD endpoint was conducted after approximately 150 participants had completed 3 months of study treatment; and 3) a third interim analysis was conducted after 450 participants had completed 6 months of study treatment. The interim analyses were performed for the purpose of internal decision making and no changes were made to the study conduct based on the results of the interim analyses.

The modified Intent-to-Treat (mITT) Population comprised all randomized participants apart from those who were randomized in error (i.e. were also recorded as screen or run-in failures and did not receive a dose of study treatment). Randomized participants were assumed to have received study medication unless definitive evidence to the contrary exists. All data summaries and analyses for this population were based on the actual treatment received, if it was different to the randomized treatment. This population constituted the primary population for all study population and safety analyses. The per protocol (PP) population comprised all participants from the mITT population who did not have a protocol deviation considered to impact efficacy.

A Bayesian dose response model of maximum observed efficacy (Emax) was used to determine the dose-response curve for the primary efficacy endpoint of this study, the change from baseline in respiratory symptoms by ERS: COPD at month 6. The dose-response model was fitted to the data using Bayesian techniques using functional uniform priors for the ED50 (dose that yields 50% of the maximal response) and m (dose-response slope) parameters and non-informative priors for E0 and Emax. The log-linear, 3-parameter Emax and 4-parameter Emax models were fitted and the best fitting model was presented. Where possible, covariates (i.e., baseline, smoking status, country) were included in the E0 and Emax terms of the selected model. This endpoint was assessed in the PP Population. Posterior mean change, standard deviation (SD), posterior median and 90% credible intervals were presented for the change from baseline for all treatment arms and all pairwise differences between each DNX dose and placebo. The posterior probability of a difference from placebo of < 0, − 0.5, − 1, − 1.5 and − 2 was presented for each DNX dose.

For secondary efficacy analyses, a Bayesian generalized linear model assuming a negative binomial distribution for the underlying exacerbation rate with a log link function was used to determine the annual rate of on-treatment moderate/severe HCRU exacerbations and EXACT events. A Bayesian proportional hazards model was used to determine time to first on-treatment moderate/severe HCRU exacerbation, time to first on-treatment severe HCRU exacerbation and time to first on-treatment EXACT event. A generalized linear mixed model was used to determine response according to E-RS:COPD total score, subscale scores, CAT score and SGRQ total score.

A frequentist mixed model repeated measures was used to analyze change from baseline post-bronchodilator FEV1 and FVC. PK parameters were calculated by standard non-compartmental analysis using Phoenix WinNonlin Version 7.0. All calculations of non-compartmental parameters were based on actual sampling times and were performed for the sub-set of participants providing serial blood samples for PK.

## Results

### Demographics

**Fig. 1:**
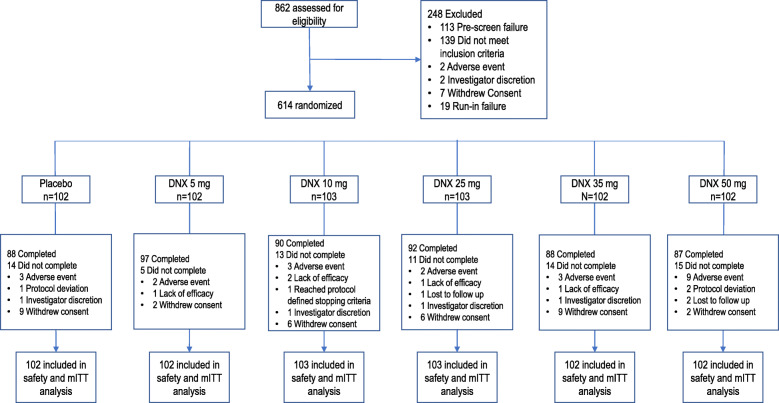
Consolidated Standards of Reporting Trials (CONSORT) flow diagram of subject disposition; mITT: modified intent-to-treat

A total of 614 participants were randomized in this study across 9 different countries (Fig. 1). Demographics and clinical characteristics of the enrolled participants are shown in Tables 1 and 2, respectively.

**Table 1:** Demographics

	Number of participants, N (%)					
	Placebo (N = 102)	DNX 5 mg (***N*** = 102)	DNX 10 mg (***N*** = 103)	DNX 25 mg (*N* = 103)	DNX 35 mg (*N* = 102)	DNX 50 mg (*N* = 102)
**Age (years)**						
Mean (SD)	66.2 (7.3)	66.3 (6.8)	65.7 (7.5)	66.3 (7.3)	65.1 (7.6)	65.7 (7.0)
Median	67.0	66.5	66.0	67.0	65.0	66.0
**Age group (years)**						
18–64	43 (42)	38 (37)	45 (44)	40 (39)	46 (45)	47 (46)
65–74	46 (45)	52 (51)	47 (46)	47 (46)	45 (44)	40 (39)
75–84	13 (13)	12 (12)	11 (11)	16 (16)	11 (11)	15 (15)
**Sex**						
Female	29 (28)	36 (35)	32 (31)	38 (37)	35 (34)	32 (31)
Male	73 (72)	66 (65)	71 (69)	65 (63)	67 (66)	70 (69)
**Ethnicity**						
Hispanic/Latino	1 (< 1)	0	1 (< 1)	3 (3)	1 (< 1)	3 (3)
Not Hispanic/Latino	101 (> 99)	102 (100)	102 (> 99)	100 (97)	101 (> 99)	99 (97)
**Race**						
Asian - East Asian Heritage	10 (10)	6 (6)	18 (17)	17 (17)	10 (10)	17 (17)
Asian - South East Asian Heritage	1 (< 1)	0	0	0	0	0
Black or African American	2 (2)	2 (2)	1 (< 1)	0	0	1 (< 1)
Native Hawaiian or other Pacific Islander	1 (< 1)	0	0	0	0	0
White - Arabic/North African Heritage	1 (< 1)	1 (< 1)	0	0	0	0
White - White/Caucasian/European Heritage	87 (85)	93 (91)	84 (82)	86 (83)	92 (90)	84 (82)

**Table 2:** Baseline Clinical Characteristics

	Number of participants, N (%)					
	Placebo(*N* = 102)	DNX 5 mg (*N* = 102)	DNX 10 mg (*N* = 103)	DNX 25 mg (*N* = 103)	DNX 35 mg (*N* = 102)	DNX 50 mg (*N* = 102)
Current smoker	39 (38)	40 (39)	37 (36)	35 (34)	38 (37)	36 (35)
Former smoker	63 (62)	62 (61)	66 (64)	68 (66)	64 (63)	66 (65)
**SGRQ**						
Q1 (I cough) most days a week or several days a week	80 (79)	86 (85)	74 (73)	80 (78)	78 (76)	82 (81)
Q2 (I bring up phlegm) most days a week or several days a week	82 (81)	80 (79)	65 (64)	81 (79)	77 (75)	80 (79)
**Chronic mucus hypersecretion (CMH)**^a^						
CMH+	75 (74)	76 (75)	58 (57)	75 (74)	68 (67)	73 (72)
CMH-	26 (26)	25 (25)	43 (43)	27 (26)	34 (33)	28 (28)
**E-RS: COPD total score**						
< 10	37 (36)	35 (34)	42 (41)	44 (43)	37 (36)	45 (44)
≥ 10	65 (64)	67 (66)	60 (59)	59 (57)	65 (64)	57 (56)
**CAT score**						
< 10	13 (13)	11 (11)	14 (14)	14 (14)	8 (8)	9 (9)
≥ 10	88 (87)	90 (89)	87 (86)	85 (86)	93 (92)	91 (91)
**Exacerbation history:**						
Moderate/severe						
1	15 (15)	19 (19)	26 (25)	17 (17)	11 (11)	22 (22)
≥ 2	87 (85)	83 (81)	76 (74)	86 (83)	91 (89)	80 (78)
**Medications**						
ICS + LABA + LAMA	39 (38)	44 (43)	45 (44)	43 (42)	39 (38)	40 (39)
LABA + LAMA	24 (24)	23 (23)	30 (29)	20 (19)	21 (21)	34 (33)
ICS + LABA	19 (19)	20 (20)	19 (18)	27 (26)	25 (25)	15 (15)
LAMA	11 (11)	7 (7)	4 (4)	8 (8)	10 (10)	5 (5)
**Inflammatory markers**						
CRP (mg/L)	5.6 (9.4)	7.2 (23.8)	7.1 (15.1)	8.0 (17.5)	4.3 (5.0)	5.5 (8.1)
Fibrinogen (g/L)	3.3 (0.9)	3.4 (1.0)	3.4 (0.9)	3.4 (0.8)	3.3 (0.8)	3.4 (0.8)

^a^A subject is considered CMH+ if baseline SGRQ Q1 and Q2 = Most or several days a week.

### Efficacy

**Fig. 2:**
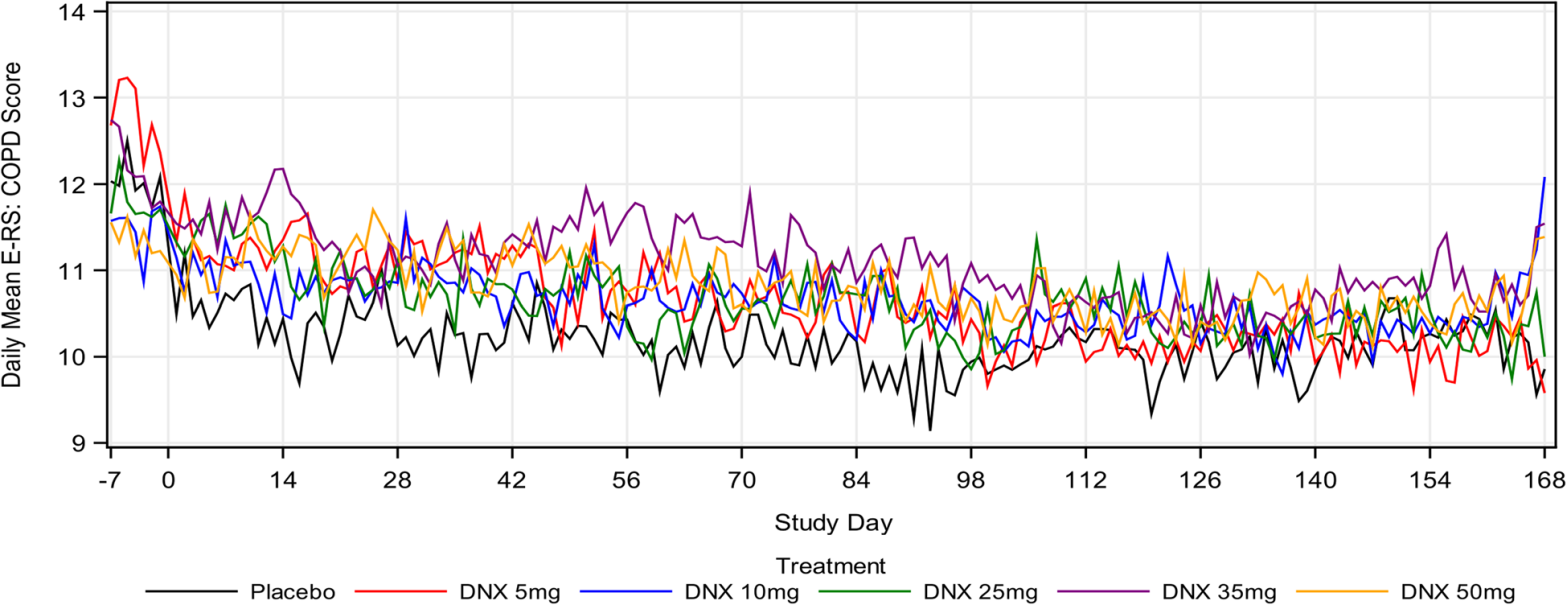
Daily mean E-RS: COPD total scores over time. Baseline ERS:COPD Total Scores, Mean (SD): Placebo = 12.01 (6.299), DNX 5 mg = 12.73 (6.232), DNX 10 mg = 11.53 (6.288), DNX 25 mg = 11.70 (6.724), DNX 35 mg = 12.08 (5.804), DNX 50 mg = 11.43 (5.219)

Baseline E-RS: COPD total score and sub-scores of breathlessness, cough and sputum, and chest symptoms were similar across all treatment groups. Over one-third of participants had a baseline ERS score lower than 10, suggesting a low symptom burden (Table 2). Adherence to diary completion was very high in the study, with 79–87% of subjects in each treatment group exhibiting > 90% daily compliance. The posterior mean change from baseline in ERS: COPD total score and sub-scores at month 6 across all treatment groups demonstrated a trend toward decreased scores, indicating an improvement in respiratory symptoms. The placebo group had the most negative mean change from baseline (− 2.11), ie the most improvement, and was the only group that achieved the proposed MCID (Fig. 2). The mean change from baseline in ERS: COPD total scores in the DNX groups diminished in a dose-dependent manner, indicating less improvement as the dose increased. Changes in the E-RS: COPD total scores appeared to be due to changes in the breathlessness and cough and sputum sub-scores, however, at month 6, 49–67% of all patients were non-responders.

The mean change from baseline in E-RS: COPD total score at months 1 to 6 across all treatment groups demonstrated a trend toward decreased scores, indicating an improvement in respiratory symptoms. Overall, the daily mean change from baseline E-RS: COPD total score and for the 3 subscales of breathlessness, cough and sputum and chest symptoms for DNX treated participants trended higher than those treated with placebo. A decrease in the E-RS: COPD total score and sub-scores was observed for all treatment groups during the run-in period (Day − 7 to Day 1), demonstrating a pronounced study effect (Fig. 2). Exploration of multiple subgroups (smoking status, lung function, CAT, SGRQ, seasonality, exacerbation history, presence of chronic mucus hypersecretion (CMH), and baseline medication use) did not identify a population that could benefit from DNX treatment (data not shown).

HCRU Exacerbations, EXACT events, SGRQ and CAT were assessed as secondary endpoints. The number of moderate-severe exacerbations in the DNX groups ranged from 56 to 75, with no evidence of a dose response, compared to 44 events in the placebo group. The time to first moderate/severe HCRU exacerbation ranged from 47 to 79 days in the DNX groups, compared to 110 days in the placebo group. Fewer EXACT events compared to HCRU exacerbations were reported, ranging from 9 to 19 in the DNX groups and 9 events in the placebo group.

At month 6, the posterior mean change from baseline in SGRQ scores ranged from − 4.94 to − 3.41; four of the six groups (placebo, DNX 10 mg, 25 mg and 35 mg) achieved the MCID for SGRQ, but there no improvement in the DNX groups compared placebo (Table 3). The percentage of responders at month 6 ranged from 40 to 52%, with no dose response. The posterior mean change from baseline in CAT scores at month 6 ranged from − 0.97 to − 1.56, with the placebo group having a mean change from baseline of − 1.39 and no evidence of a dose response for the DNX groups. The percentage of responders at month 6 ranged from 45 to 54%, with no dose response (Table 4).

**Table 3:** Bayesian Analysis of Change from Baseline SGRQ Score up to Month 6

	Placebo	DNX 5 mg	DNX 10 mg	DNX 25 mg	DNX 35 mg	DNX 50 mg
**N**	101	102	100	103	100	99
**Baseline SGRQ Total Score**	46.21 (17.426)	47.16 (16.057)	45.97 (14.991)	48.47 (17.514)	47.18 (15.871)	46.19 (16.669)
**n**	85	96	86	90	86	85
**Mean Change from Baseline (90% CI)**	−4.11 (−6.25,-2.00)	−3.44 (−5.51,-1.38)	−4.19 (−6.28,-2.12)	−4.94 (−7.03,-2.91)	−4.12 (−6.22,-1.99)	−3.41 (−5.55,-1.26)
**Mean Difference from Placebo (90% CI)**		0.68 (−2.26,3.67)	−0.08 (− 3.05,2.84)	−0.83 (− 3.81,2.09)	−0.01 (− 3.06,2.97)	0.70 (−2.33,3.76)

*N* Number enrolled, *n* Number of subjects with analysable data at the current time point; *CI* Credible interval

**Table 4:** Bayesian Analysis of Change from Baseline CAT Score up to Month 6

	Placebo	DNX 5 mg	DNX 10 mg	DNX 25 mg	DNX 35 mg	DNX 50 mg
**N**	101	102	100	103	100	99
**Baseline CAT Total Score**	18.2 (7.72)	18.3 (7.11)	17.8 (6.99)	18.2 (7.80)	19.1 (6.74)	17.5 (6.20)
**n**	84	94	86	87	85	83
**Mean Change from Baseline (90% CI)**	−1.39 (−2.29,-0.47)	−1.39 (− 2.27,-0.51)	−1.23 (− 2.13,-0.32)	−0.97 (−1.91,-0.04)	−1.56 (− 2.47,-0.64)	− 1.32 (− 2.24,-0.39)
**Mean Difference from Placebo (90% CI)**		−0.01 (− 1.29,1.29)	0.16 (− 1.12,1.46)	0.41 (− 0.88,1.69)	−0.17 (− 1.48,1.10)	0.07 (− 1.26,1.37)

*N* Number enrolled, *n* Number of subjects with analysable data at the current time point; *CI* Credible interval

Lung function and rescue medication use remained stable over the course of the study within each treatment group and was similar between groups. No improvement was observed in the DNX-treated groups compared to placebo. Complete data were available from 50 participants in the physical activity sub-study. Baseline PROactive total scores ranged from 59.63–68.97. The mean change from baseline at month 6 in the placebo group was − 0.96 and ranged from 0.43–4.08 in the DNX groups with no evidence of a dose response.

### Safety

The overall incidence of on-treatment AEs was similar between the DNX 5 mg to 35 mg groups (range: 62 to 67%) compared with Placebo (62%) (Table S1). The slightly higher incidence of AEs seen in the DNX 50 mg group (70%) was not related to a specific term or category. There appeared to be a dose-related increase in the incidence of events in the nervous system disorders category in the DNX groups, likely due to increased incidence of headache.

The most frequently reported on-treatment AE for all treatment groups was nasopharyngitis, the incidence of which was similar across all treatment groups (Table 5). The incidence of drug-related on-treatment AEs was similar in the DNX groups compared with Placebo with no DNX dose-related trend (Table S2). The incidence of pneumonia was higher in the DNX 50 mg group (5%) compared with both Placebo (< 1%) and the remaining DNX groups (range: < 1 to 2%). Of the 12 on-treatment pneumonia events, almost all (10/12) were reported for participants who took ICS for > 7 days prior to the time of pneumonia onset. No participant with a pneumonia event had evidence of decreased neutrophil counts or neutropenia. The incidence of all remaining on-treatment AEs was generally similar in the DNX and Placebo groups.

**Table 5:** Most Frequently Reported On-Treatment AEs (≥5% Incidence in Any Treatment Group)

	Number of participants, n (%)					
Adverse Event (Preferred term)	Placebo N = 102	DNX 5 mg N = 102	DNX 10 mg N = 103	DNX 25 mg N = 103	DNX 35 mg ***N*** = 102	DNX 50 mg N = 102
Any event	63 (62)	63 (62)	69 (67)	68 (66)	63 (62)	71 (70)
Nasopharyngitis	12 (12)	8 (8)	9 (9)	12 (12)	14 (14)	10 (10)
Upper respiratory tract infection	5 (5)	7 (7)	7 (7)	9 (9)	5 (5)	6 (6)
Back pain	4 (4)	7 (7)	4 (4)	6 (6)	5 (5)	5 (5)
Headache	2 (2)	4 (4)	6 (6)	5 (5)	8 (8)	8 (8)
Arthralgia	2 (2)	0	5 (5)	1 (< 1)	2 (2)	4 (4)
Cough	1 (< 1)	4 (4)	1 (< 1)	2 (2)	1 (< 1)	5 (5)
Pneumonia	1 (< 1)	1 (< 1)	2 (2)	2 (2)	1 (< 1)	5 (5)

The incidence of on-treatment serious AEs (SAEs) was similar in the DNX groups compared with placebo with no dose-related trend (Table S3). All SAEs of pneumonia were reported in the DNX groups, and the highest incidence occurred in the DNX 50 mg group (4%). Two participants in the DNX 50 mg group withdrew due to the pneumonia SAE, one of which was considered drug-related by the investigator. There were 5 on-treatment fatal SAEs reported in this study, all of which occurred in the DNX treatment groups (1 in DNX 10 mg, 2 in DNX 25 mg, 1 in DNX 35 mg, and 1 in DNX 50 mg). None of these events was considered drug-related by the investigator. One death was reported as due to septic shock (DNX 50 mg group) and the remaining were reported as being of unknown cause.

There were no significant differences in hematology, clinical chemistry values, vital signs or ECG parameters between placebo and the DNX groups. Neutrophil counts remained stable in participants receiving DNX, and no participant had a reported finding of neutropenia (Fig. S1). One participant receiving DNX 10 mg with baseline transaminases approximately 1.5-2x the upper limit of normal (ULN) met the protocol-defined liver stopping criteria during the study. Maximal elevations of ALT (7x ULN) and AST (11x ULN) were observed on Day 84, with normal bilirubin. Study treatment was discontinued, and transaminases returned to baseline within 10 days.

### Pharmacokinetics

Concentration-time profiles showed that DNX blood concentrations increased with increasing doses of DNX (Table S4). Systemic exposure as measured by Cmax and AUC(0-t) was characterized by generally moderate to high between-participant variability (%CVb) for all doses of DNX. There was no appreciable effect of age, weight or gender on individual whole blood PK parameters, Cmax and AUC(0-t) (data not shown).

### Biomarkers

There were no changes in serum CRP or plasma fibrinogen (data not shown). An analysis of 7 exploratory serum biomarkers reflective of extracellular matrix synthesis and degradation, EL-NE, PRO-C4, PRO-C6, C6M, ELP-3, EL-CG and C4Ma3, collected at baseline, month 3 and month 6, demonstrated no difference between any of the treatment groups (data not shown).

## Discussion

This 6-month study evaluated the effect of 5 doses of DNX (5, 10, 25, 35, and 50 mg) compared to placebo in symptomatic patients with COPD who had at least one moderate/severe exacerbation in the past 12 months. Treatment with DNX did not demonstrate any clinically meaningful benefit on COPD symptoms (ERS: COPD) or health-related quality of life (SGRQ) and exploration of multiple subgroups did not identify a population that could benefit from DNX treatment, contrary to an earlier study that suggested COPD patients who were current smokers might benefit from treatment with a CXCR2 antagonist [25]. Treatment with DNX was associated with more exacerbations and a higher frequency of pneumonia-related events. Both lack of efficacy and unfavorable side-effects were unexpected based on the phase 2a data published recently [7].

While most patients across all treatment groups had a baseline E-RS: COPD score ≥ 10, characteristic of a symptomatic COPD population, a substantial proportion of subjects (34–44%) had ERS: COPD scores < 10, suggesting a low or absent symptom burden. Most patients had CAT scores ≥10 and had a history of 2 or more moderate/severe exacerbations over the past 12 months. Given that approximately 40% of participants had a minimal symptom burden despite the requirement for current respiratory symptoms to be included in the study, it could be argued that the study population was not entirely appropriate for assessing the primary endpoint of E-RS:COPD.

A possible contributor to the lack of benefit is the large placebo response. The magnitude of the placebo response was an unexpected finding and was not observed in recent studies utilizing this symptom diary [26, 27] or in a prior danirixin clinical study in a similar population [7]. The previous Phase 2a study was performed in a smaller number of participants in one country with more consistent healthcare, thereby potentially minimizing the placebo effect in that study. Furthermore, during the 7-day run-in period prior to treatment, a study effect was observed in the form of improvement in daily mean E-RS: COPD total score and subscales for all treatment groups including placebo. It is unclear whether this study effect was due to greater adherence to daily medication or as a result of greater awareness of personal health state due to daily completion of a diary. There was no requirement for study participants to be on specific maintenance therapy but rather, the protocol allowed investigators to use clinical judgement to appropriately manage their patients. The sample size of the study was insufficient to determine if different maintenance therapies may have had an impact on the activity of danirixin. A final possibility is the inherent variability of subjective endpoints in COPD patients; a recent analysis of a large COPD cohort that measured repeatability of measurements within a 6-week period demonstrated that only 44 and 25% of COPD patients reported no change in SGRQ and CAT scores, respectively [28].

The key safety risk for danirixin, based on the mechanism of action, was the potential to impact host defense. We observed an increase in the number of study participants developing on-treatment pneumonia with the highest dose of DNX (50 mg). In addition, the overall incidence of adverse events in the Infections category was higher in the DNX treatment groups compared to placebo and treatment was associated with a higher number of exacerbations and a shorter time to first exacerbation. These effects could not readily be attributed to an overt change in neutrophil number, as peripheral blood neutrophil counts remained stable throughout the study, but possibly reflects changes in neutrophil trafficking and function. An increased number of pneumonia events was unexpected as this was not observed in a prior clinical study of 1-year duration, although the exposure at 50 mg in the current study using the hydrobromide salt formulation was higher than previously achieved using the free base formulation [7, 13].

## Conclusions

The robust placebo response and study effects prohibited any conclusions on the efficacy of DNX. Lengthening the run-in period may have allowed for a more stabilized patient population and controlled for the observed study effect. In addition, the use of a disease impact score such as CAT as part of the entry criteria may have ensured a more appropriate study population. However, the absence of a clear efficacy benefit and the observed increase in exacerbations in DNX-treated groups suggests an unfavorable benefit-risk profile for danirixin in patients with COPD.

## Supplementary information

**Additional file 1:** Additional safety and pharmacokinetic data.

## Data Availability

Anonymized individual participant data and study documents can be requested for further research from www.clinicalstudydatarequest.com.

## Abbreviations

AE: Adverse event
CAT: COPD Assessment test
CMH: Chronic mucus hypersecretion
COPD: Chronic Obstructive Pulmonary Disease
CPPAC: Clinic Visit PROactive Physical Activity in COPD
CXCR2: CXC chemokine receptor 2
DNX: Danirixin
Emax: Maximum observed efficacy
E-RS: COPD: Evaluating Respiratory Symptoms in COPD
EXACT: EXAcerbations of Chronic Pulmonary Disease Tool
FEV1: Forced expiratory volume in one second
FVC: Forced vital capacity
HCRU: Healthcare resource utilization
HRQoL: Health-related Quality of Life
MCID: Minimal clinically important difference
mITT: Modified Intent-to-Treat
PRO: Patient reported outcome
SAE: Serious adverse event
SD: Standard deviation
SGRQ-C: St. George Respiratory Questionnaire-COPD
ULN: Upper limit of normal
