# Supplementary material for: CXCR2 antagonist for patients with chronic obstructive pulmonary disease with chronic mucus hypersecretion: a phase 2b trial
Source: Respir Res. 2020 Jun 12;21:149. doi: 10.1186/s12931-020-01401-4 (PMC7291447; doi:10.1186/s12931-020-01401-4)
Supplement: Supplementary file 1 — Additional file 1: Additional safety and pharmacokinetic data. [file 12931_2020_1401_MOESM1_ESM.docx]

Contents

Table S1: On-Treatment AEs by SOC

Table S2: Drug-Related On-Treatment Adverse Events

Table S3: On-Treatment SAEs

Table S4: Danirixin Steady State Whole Blood Pharmacokinetic Parameters (Day 168)

Figure S1: Mean (95%CI) Neutrophil Counts over Time

Table S1 On-Treatment AEs by SOC

|  | **Number of participants, n (%)** | | | | | |
| --- | --- | --- | --- | --- | --- | --- |
| **System Organ Class** | **Placebo**  **N=102** | **DNX**  **5 mg**  **N=102** | **DNX**  **10 mg**  **N=103** | **DNX**  **25 mg**  **N=103** | **DNX**  **35 mg**  **N=102** | **DNX**  **50 mg**  **N=102** |
| Any event | 63 (62) | 63 (62) | 69 (67) | 68 (66) | 63 (62) | 71 (70) |
| Infections and infestations | 29 (28) | 35 (34) | 28 (27) | 36 (35) | 34 (33) | 37 (36) |
| Gastrointestinal disorders | 14 (14) | 14 (14) | 13 (13) | 15 (15) | 13 (13) | 14 (14) |
| Musculoskeletal and connective tissue disorders | 9 (9) | 12 (12) | 18 (17) | 15 (15) | 14 (14) | 14 (14) |
| Nervous system disorders | 8 (8) | 6 (6) | 9 (9) | 14 (14) | 10 (10) | 16 (16) |
| Respiratory, thoracic and mediastinal disorders | 7 (7) | 14 (14) | 12 (12) | 9 (9) | 10 (10) | 18 (18) |
| Metabolism and nutrition disorders | 6 (6) | 5 (5) | 4 (4) | 5 (5) | 5 (5) | 2 (2) |
| Vascular disorders | 6 (6) | 3 (3) | 3 (3) | 2 (2) | 6 (6) | 4 (4) |
| Injury, poisoning and procedural complications | 4 (4) | 4 (4) | 2 (2) | 6 (6) | 2 (2) | 6 (6) |
| Neoplasms benign, malignant and unspecified (including cysts and polyps) | 4 (4) | 2 (2) | 2 (2) | 2 (2) | 1 (<1) | 2 (2) |
| General disorders and administration site conditions | 3 (3) | 6 (6) | 9 (9) | 6 (6) | 6 (6) | 10 (10) |
| Cardiac disorders | 3 (3) | 5 (5) | 3 (3) | 3 (3) | 1 (<1) | 2 (2) |
| Skin and subcutaneous tissue disorders | 1 (<1) | 4 (4) | 4 (4) | 5 (5) | 5 (5) | 2 (2) |
| Investigations | 1 (<1) | 3 (3) | 6 (6) | 5 (5) | 2 (2) | 3 (3) |
| Ear and labyrinth disorders | 1 (<1) | 3 (3) | 3 (3) | 1 (<1) | 2 (2) | 2 (2) |
| Psychiatric disorders | 1 (<1) | 1 (<1) | 0 | 0 | 7 (7) | 2 (2) |
| Immune system disorders | 1 (<1) | 0 | 0 | 1 (<1) | 0 | 0 |
| Blood and lymphatic system disorders | 0 | 3 (3) | 1 (<1) | 3 (3) | 1 (<1) | 0 |
| Eye disorders | 0 | 2 (2) | 2 (2) | 0 | 3 (3) | 1 (<1) |
| Renal and urinary disorders | 0 | 1 (<1) | 3 (3) | 1 (<1) | 2 (2) | 4 (4) |
| Hepatobiliary disorders | 0 | 0 | 1 (<1) | 1 (<1) | 0 | 2 (2) |
| Reproductive system and breast disorders | 0 | 0 | 0 | 0 | 1 (<1) | 1 (<1) |

**Table S2 Drug-Related On-Treatment Adverse Events**

|  | **Number of participants, n (%)** | | | | | |
| --- | --- | --- | --- | --- | --- | --- |
| **Adverse Event**  **(Preferred term)** | **Placebo**  **N=102** | **DNX**  **5 mg**  **N=102** | **DNX**  **10 mg**  **N=103** | **DNX**  **25 mg**  **N=103** | **DNX**  **35 mg**  **N=102** | **DNX**  **50 mg**  **N=102** |
| Any event | 7 (7) | 12 (12) | 8 (8) | 7 (7) | 10 (10) | 8 (8) |
| Diarrhoea | 3 (3) | 2 (2) | 1 (<1) | 2 (2) | 1 (<1) | 2 (2) |
| Nasopharyngitis | 0 | 1 (<1) | 0 | 0 | 2 (2) | 0 |
| Abdominal pain | 0 | 1 (<1) | 0 | 0 | 0 | 1 (<1) |
| Abdominal pain upper | 1 (<1) | 0 | 0 | 0 | 0 | 1 (<1) |
| Arthralgia | 1 (<1) | 0 | 0 | 0 | 0 | 1 (<1) |
| Dizziness | 0 | 0 | 1 (<1) | 1 (<1) | 0 | 0 |
| Fatigue | 1 (<1) | 0 | 0 | 0 | 1 (<1) | 0 |
| Headache | 0 | 1 (<1) | 0 | 1 (<1) | 0 | 0 |
| Insomnia | 0 | 0 | 0 | 0 | 1 (<1) | 1 (<1) |
| Musculoskeletal stiffness | 0 | 0 | 0 | 1 (<1) | 1 (<1) | 0 |
| Nausea | 1 (<1) | 0 | 0 | 1 (<1) | 0 | 0 |
| Pneumonia | 0 | 1 (<1) | 0 | 0 | 0 | 1 (<1) |
| Abnormal faeces | 0 | 0 | 0 | 0 | 0 | 1 (<1) |
| Aspartate aminotransferase increased | 0 | 0 | 0 | 1 (<1) | 0 | 0 |
| Atrioventricular block first degree | 1 (<1) | 0 | 0 | 0 | 0 | 0 |
| Blood pressure increased | 0 | 0 | 1 (<1) | 0 | 0 | 0 |
| Cardiac failure congestive | 0 | 0 | 0 | 1 (<1) | 0 | 0 |
| Conjunctival haemorrhage | 0 | 0 | 1 (<1) | 0 | 0 | 0 |
| Conjunctivitis | 0 | 1 (<1) | 0 | 0 | 0 | 0 |
| Constipation | 0 | 0 | 0 | 0 | 0 | 1 (<1) |
| Contusion | 0 | 1 (<1) | 0 | 0 | 0 | 0 |
| Cough | 0 | 0 | 0 | 0 | 1 (<1) | 0 |
| Decreased appetite | 1 (<1) | 0 | 0 | 0 | 0 | 0 |
| Dyspepsia | 0 | 0 | 0 | 0 | 0 | 1 (<1) |
| Epistaxis | 0 | 0 | 0 | 0 | 0 | 1 (<1) |
| Faeces discoloured | 0 | 0 | 0 | 0 | 1 (<1) | 0 |
| Gastric disorder | 0 | 0 | 0 | 1 (<1) | 0 | 0 |
| Gastroenteritis | 0 | 1 (<1) | 0 | 0 | 0 | 0 |
| Gastroenteritis bacterial | 0 | 1 (<1) | 0 | 0 | 0 | 0 |
| Haemoptysis | 0 | 0 | 1 (<1) | 0 | 0 | 0 |
| Hot flush | 1 (<1) | 0 | 0 | 0 | 0 | 0 |
| Hypertonic bladder | 0 | 0 | 0 | 1 (<1) | 0 | 0 |
| Muscular weakness | 0 | 0 | 1 (<1) | 0 | 0 | 0 |
| Nasal congestion | 0 | 0 | 0 | 0 | 1 (<1) | 0 |
| Nasal obstruction | 0 | 0 | 0 | 0 | 0 | 1 (<1) |
| Neutrophilia | 0 | 1 (<1) | 0 | 0 | 0 | 0 |
| Ocular hypertension | 0 | 0 | 0 | 0 | 1 (<1) | 0 |
| Oedema peripheral | 0 | 0 | 1 (<1) | 0 | 0 | 0 |
| Oropharyngeal pain | 0 | 1 (<1) | 0 | 0 | 0 | 0 |
| Pain in extremity | 0 | 0 | 0 | 0 | 0 | 1 (<1) |
| Productive cough | 0 | 0 | 0 | 0 | 0 | 1 (<1) |
| Prostatic disorder | 0 | 0 | 0 | 0 | 1 (<1) | 0 |
| Pruritus | 0 | 0 | 1 (<1) | 0 | 0 | 0 |
| Purpura | 0 | 0 | 1 (<1) | 0 | 0 | 0 |
| Rash | 0 | 0 | 0 | 1 (<1) | 0 | 0 |
| Rash generalised | 0 | 1 (<1) | 0 | 0 | 0 | 0 |
| Rhinorrhoea | 0 | 1 (<1) | 0 | 0 | 0 | 0 |
| Salivary hypersecretion | 1 (<1) | 0 | 0 | 0 | 0 | 0 |
| Sinonasal obstruction | 0 | 1 (<1) | 0 | 0 | 0 | 0 |
| Somnolence | 0 | 0 | 0 | 0 | 0 | 1 (<1) |
| Thirst | 0 | 0 | 0 | 0 | 0 | 1 (<1) |
| Tonsillitis | 0 | 0 | 0 | 0 | 1 (<1) | 0 |
| Vomiting | 1 (<1) | 0 | 0 | 0 | 0 | 0 |
| Weight increased | 0 | 1 (<1) | 0 | 0 | 0 | 0 |
|  | | | | | | |

**Table S3 On-Treatment SAEs**

|  | **Number of participants, n (%)** | | | | | |
| --- | --- | --- | --- | --- | --- | --- |
| **SAE**  **(Preferred term)** | **Placebo**  **N=102** | **DNX**  **5 mg**  **N=102** | **DNX**  **10 mg**  **N=103** | **DNX**  **25 mg**  **N=103** | **DNX**  **35 mg**  **N=102** | **DNX**  **50 mg**  **N=102** |
| Any event | 8 (8) | 7 (7) | 13 (13) | 10 (10) | 7 (7) | 11 (11) |
| Pneumonia | 0 | 0 | 2 (2) | 1 (<1) | 1 (<1) | 4 (4) |
| COPD | 1 (<1) | 1 (<1) | 1 (<1) | 2 (2) | 2 (2) | 0 |
| Atrial fibrillation | 0 | 1 (<1) | 2 (2) | 0 | 0 | 0 |
| Death | 0 | 0 | 1 (<1) | 2 (2) | 0 | 0 |
| Osteoarthritis | 1 (<1) | 0 | 1 (<1) | 1 (<1) | 0 | 0 |
| Angina pectoris | 1 (<1) | 1 (<1) | 0 | 0 | 0 | 0 |
| Bladder cancer | 0 | 1 (<1) | 1 (<1) | 0 | 0 | 0 |
| Lung neoplasm malignant | 0 | 0 | 1 (<1) | 1 (<1) | 0 | 0 |
| Anaemia | 0 | 0 | 0 | 1 (<1) | 0 | 0 |
| Anal prolapse | 0 | 0 | 1 (<1) | 0 | 0 | 0 |
| Ankylosing spondylitis | 0 | 0 | 0 | 0 | 0 | 1 (<1) |
| Arthritis bacterial | 0 | 0 | 0 | 0 | 0 | 1 (<1) |
| Bladder papilloma | 0 | 0 | 0 | 0 | 0 | 1 (<1) |
| Cardiac neoplasm unspecified | 1 (<1) | 0 | 0 | 0 | 0 | 0 |
| Chronic gastritis | 1 (<1) | 0 | 0 | 0 | 0 | 0 |
| Coronary artery disease | 0 | 0 | 1 (<1) | 0 | 0 | 0 |
| Decreased appetite | 1 (<1) | 0 | 0 | 0 | 0 | 0 |
| Deep vein thrombosis | 0 | 0 | 0 | 1 (<1) | 0 | 0 |
| Diabetes mellitus | 0 | 0 | 0 | 1 (<1) | 0 | 0 |
| Diabetic neuropathy | 0 | 0 | 0 | 1 (<1) | 0 | 0 |
| Epistaxis | 0 | 0 | 0 | 0 | 0 | 1 (<1) |
| Facial bones fracture | 0 | 0 | 0 | 0 | 0 | 1 (<1) |
| Faecaloma | 0 | 0 | 0 | 1 (<1) | 0 | 0 |
| Gastrointestinal perforation | 0 | 1 (<1) | 0 | 0 | 0 | 0 |
| Gastrooesophageal reflux disease | 0 | 0 | 1 (<1) | 0 | 0 | 0 |
| Haematuria | 0 | 0 | 0 | 0 | 0 | 1 (<1) |
| Haemoptysis | 0 | 0 | 1 (<1) | 0 | 0 | 0 |
| Haemorrhoids | 0 | 0 | 1 (<1) | 0 | 0 | 0 |
| Hemiparesis | 0 | 0 | 0 | 0 | 0 | 1 (<1) |
| Hepatic cyst | 0 | 0 | 0 | 0 | 0 | 1 (<1) |
| Hepatic steatosis | 0 | 0 | 0 | 1 (<1) | 0 | 0 |
| Ischaemic cardiomyopathy | 0 | 0 | 0 | 0 | 1 (<1) | 0 |
| Large intestine perforation | 0 | 0 | 0 | 1 (<1) | 0 | 0 |
| Large intestine polyp | 1 (<1) | 0 | 0 | 0 | 0 | 0 |
| Lower gastrointestinal haemorrhage | 0 | 0 | 1 (<1) | 0 | 0 | 0 |
| Lymphadenopathy | 0 | 0 | 0 | 1 (<1) | 0 | 0 |
| Metapneumovirus infection | 0 | 1 (<1) | 0 | 0 | 0 | 0 |
| Mitral valve incompetence | 0 | 1 (<1) | 0 | 0 | 0 | 0 |
| Musculoskeletal chest pain | 0 | 0 | 0 | 0 | 0 | 1 (<1) |
| Myocardial infarction | 0 | 1 (<1) | 0 | 0 | 0 | 0 |
| Oesophageal adenocarcinoma | 1 (<1) | 0 | 0 | 0 | 0 | 0 |
| Pancreatitis | 0 | 0 | 0 | 0 | 1 (<1) | 0 |
| Pancreatitis chronic | 0 | 0 | 0 | 1 (<1) | 0 | 0 |
| Perichondritis | 0 | 0 | 0 | 0 | 0 | 1 (<1) |
| Pilonidal cyst | 0 | 0 | 1 (<1) | 0 | 0 | 0 |
| Prostate cancer | 1 (<1) | 0 | 0 | 0 | 0 | 0 |
| Psoas abscess | 0 | 1 (<1) | 0 | 0 | 0 | 0 |
| Pulmonary embolism | 0 | 0 | 0 | 1 (<1) | 0 | 0 |
| Pyelonephritis acute | 0 | 0 | 0 | 0 | 0 | 1 (<1) |
| Sciatica | 0 | 0 | 0 | 0 | 0 | 1 (<1) |
| Septic shock | 0 | 0 | 0 | 0 | 0 | 1 (<1) |
| Small intestinal obstruction | 0 | 0 | 1 (<1) | 0 | 0 | 0 |
| Squamous cell carcinoma | 0 | 0 | 0 | 0 | 1 (<1) | 0 |
| Sudden death | 0 | 0 | 0 | 0 | 1 (<1) | 0 |
| Vertigo | 0 | 0 | 0 | 1 (<1) | 0 | 0 |

**Table S4 Danirixin Steady State Whole Blood Pharmacokinetic Parameters (Day 168)**

| **PK Parameter**  **(units)** | **Treatment** | **N** | **n^2^** | **Geometric Mean (%CVb)** | **95% CI** |
| --- | --- | --- | --- | --- | --- |
| **Cmax**  **(ng/mL)** | **DNX 5 mg** | 102 | 14 | 171.9 (62.36) | (123.5, 239.4) |
|  | **DNX 10 mg** | 103 | 13 | 357.3 (45.89) | (274.4, 465.4) |
|  | **DNX 25 mg** | 102 | 17 | 821.2 (73.40) | (570.9, 1181.2) |
|  | **DNX 35 mg** | 102 | 18 | 1695.0 (60.15) | (1285.8, 2234.5) |
|  | **DNX 50 mg** | 101 | 16 | 2390.5 (32.95) | (2014.6, 2836.5) |
| **tmax^1^**  **(h)** | **DNX 5 mg** | 102 | 14 | 1.000 | (0.50-11.78) |
|  | **DNX 10 mg** | 103 | 13 | 1.000 | (0.50-2.00) |
|  | **DNX 25 mg** | 102 | 15 | 1.000 | (0.33-10.00) |
|  | **DNX 35 mg** | 102 | 18 | 1.000 | (0.48-11.87) |
|  | **DNX 50 mg** | 101 | 16 | 1.542 | (0.50-11.77) |
| **AUC(0-t)**  **(h*ng/mL)** | **DNX 5 mg** | 102 | 14 | 752.1 (59.69) | (546.8, 1034.4) |
|  | **DNX 10 mg** | 103 | 13 | 1701.8 (53.42) | (1257.2, 2303.7) |
|  | **DNX 25 mg** | 102 | 17 | 4170.1 (50.81) | (3198.1, 5437.6) |
|  | **DNX 35 mg** | 102 | 18 | 7682.6 (38.53) | (6384.8, 9244.0) |
|  | **DNX 50 mg** | 101 | 16 | 11538.0 (41.88) | (9313.4, 14294.0) |
| **tlast^1^**  **(h)** | **DNX 5 mg** | 102 | 14 | 11.900 | (4.00-12.00) |
|  | **DNX 10 mg** | 103 | 13 | 11.833 | (8.00-12.00) |
|  | **DNX 25 mg** | 102 | 15 | 11.833 | (11.75-12.00) |
|  | **DNX 35 mg** | 102 | 18 | 11.908 | (10.88-12.00) |
|  | **DNX 50 mg** | 101 | 16 | 11.933 | (4.00-12.00) |

1. Median (range) presented for tmax and tlast
2. number of subjects with data for PK parameter at the relevant visit.

**Figure S1 Mean (95%CI) Neutrophil Counts over Time**
